# Classification of audiograms in the prevention of noise-induced hearing loss: A clinical perspective

**DOI:** 10.4102/sajcd.v67i2.691

**Published:** 2020-03-03

**Authors:** Zumbi Musiba

**Affiliations:** 1 Sustainable Communities, Barrick Gold Corporation, Dar es Salaam, Tanzania

**Keywords:** ONIHL, Audiogram classification, Hearing surveillance, Threshold shift, UKHSE categorization scheme, Audiometry, Hierarchy of controls

## Abstract

**Background:**

Noise induced hearing loss (NIHL) is a major contributor to disabling hearing loss. Engineering controls are superior to hearing protection devices (HPDs) in prevention of occupational noise induced hearing loss (ONIHL), although the latter are more commonly used. Effective use of audiometry requires quick categorization of audiograms. The UK Health and Safety Executive (UKHSE) scheme for the categorization of audiograms is a tool that accomplishes this.

**Objectives:**

The objective of this paper is to provide an overview of the classification of audiograms and build a case for the preferential use of the UKHSE’s scheme to achieve this.

**Method:**

The author provides a literature review of methods of classification for audiograms and uses a case study in a Tanzanian mining company to demonstrate how the UKHSE scheme was successfully used to enhance the existing hearing protection program.

**Results:**

The literature review identified several methods of classification based on a variation of threshold shifts from baseline. The difference was in the frequency and level of threshold shift used to determine hearing loss, and the recommended course of action once hearing loss is detected. The UKHSE scheme is simple and provides guidance on steps to be taken thereafter. This was demonstrated in a case study among miners in a mining company in Tanzania.

**Conclusion:**

The UKHSE audiogram classification scheme has the advantage of providing a straightforward, easy to determine classification that allows for intervention appropriate to the findings.

## Introduction

Occupational noise-induced hearing loss (ONIHL) contributes significantly to the burden of disabling hearing loss (Nelson, Nelson, Concha-Barrientos, & Fingernut, 2005). The mechanisms by which noise causes hearing loss and the available options for prevention have been studied extensively and have been well known for decades (Morata & Meinke, 2016). Interestingly, the prevalence of ONIHL continues to rise despite the knowledge available for its prevention. The rise is most prominent in low- and middle-income (LAMI) countries and may be related to industrialisation (Nyarubeli et al., 2019). It is well known that the best way to prevent ONIHL is to follow the hierarchy of controls with elimination, substitution or engineering methods of controlling noise at the source being more effective than methods further down the hierarchy such as use of hearing protection devices (HPDs). Although the principle of the hierarchy of controls is widely accepted, there are few, if any, studies that have evaluated the impact of engineering controls on the reduction of noise exposure and ultimately ONIHL (Morata & Meinke, 2016). There is, however, a plethora of studies that have examined the efficacy of HPDs and hearing surveillance; two methods lower down, if not at the bottom of, the hierarchy of controls. This trend of hierarchy reversal has its roots in the significant challenges with instituting controls higher up the ladder (Moroe, Khoza-Shangase, Madahana, & Nyandoro, 2019). Because HPDs seem to be the bedrock of hearing conservation programmes (HCPs), this article seeks to provide an overview on classifications of audiograms, with a case built for the use of a specific classification – the UK Health and Safety Executive (UKHSE) scheme for the categorisation of audiograms as a valuable tool for the early recognition and early intervention for protection from ONIHL.

## Literature review

Noise-induced hearing loss (NIHL) is prevalent in many industries on the African continent and the world at large. Historically, there have been very few studies conducted on ONIHL in African countries, as priority was given to primary healthcare issues. Over the years, data have been collected on this subject as occupational health practice begins to take root in areas where it did not previously exist (Stevens et al., 2011). The prevalence of ONIHL seems to be dependent on the method used to define it. The methods used to define ONIHL vary from the use of the classical boiler makers’ notch to the use of average hearing thresholds at the frequencies most affected by noise (Regis, Crispim, & Ferriera, 2014). In Ghana and Tanzania, there have been studies conducted in the mining, textiles and steel industries. The results were rather alarming as the prevalence rates were high and seemed to affect a fairly young age group. These studies include the one conducted in Tanzania among miners where the prevalence of ONIHL was 47% with the youngest age group of 20–29 years being most affected (Musiba, 2015). In another study among textile workers in Tanzania, the prevalence of NIHL was found to be 58.8% (Abraham, Massawe, Ntunaguzi, Kahinga, & Mawala, 2019). A cross-sectional study among Tanzanian steel workers revealed a prevalence of ONIHL of 48% (Nyarubeli, Tungu, Moen, & Bratveit, 2019). In Ghana, a study conducted among gold mine workers showed a prevalence of 23% (Amedofu, 2002). The huge burden of ONIHL and the bid for industrialisation in most LAMI countries such as Tanzania requires comprehensive HCPs that adequately protect workers’ hearing.

The term ‘reasonably practicable’ is often used in safety science to denote the need for a risk–benefit analysis for using a particular control or intervention. This practice translates to whether or not the management of a company, with advice from relevant safety professionals, sees the benefit of instituting the control as far outweighing the risk of not doing so. Because of the cost and technical difficulties, compounded by the time limit factor in mining, the industry heavily relies on the use of HPDs (Fausti, Wilmington, Helt, Helt, & Martin, 2005). Given the pressure to deliver results in a cost-effective manner, employers are more likely to lean towards the cheaper and less effective control measures further down the hierarchy of controls, such as HPDs (Suter, 2012). This phenomenon, which has great significance in relation to ONIHL, has also been observed in the construction industry in Australia (Kosny et al., 2016). Morata and Meinke (2016), in their review of HCPs, could not find any papers addressing the effectiveness of engineering controls. The reviewed publications mostly dealt with the use of HPDs, training on the recognition of ONIHL hazards and on the proper use of HPDs and hearing surveillance (Morata & Meinke, 2016).

Given the heavy reliance on HPDs, audiometry presents a unique opportunity to identify at-risk individuals or those who already suffer from ONIHL. One of the benefits specific to the work context is that of preventing the condition or protecting a non-suspecting ONIHL worker from the hazards of being unable to hear warning sirens or signals (McBride, 2004). The value of audiometry lies in the interpretation of the results and actions taken thereafter, without which it is a compliance tick in the box with very little benefit to the workforce.

There are a number of audiogram interpretation or classification options available – all having their associated advantages and disadvantages. Unfortunately, most of them are geared towards compensation for hearing impairment. In South Africa, the percentage loss in hearing (PLH) has previously been used for the purposes of hearing conservation and compensation (Bronkhorst & Schutte, 2013). The method requires a baseline audiogram which ‘serves as a reference value’ for comparison with periodic measurements to determine loss in hearing. Much as this method is effective when used competently, the requirement of a baseline audiogram is challenging, given that labour in the most at-risk industries is often mobile and workers rarely have access to their baseline audiograms. Furthermore, the calculations required may preclude classification of audiograms in busy centres. The method is now solely used for compensation and a new regulation based on standard threshold shift (STS) has been adopted. The regulation 839 issued by the Department of Mineral Resources advocates the use of STS based on a ‘mile stone baseline’. The mile stone baseline is described as a current audiogram taken under prescribed conditions which serves as the audiometric zero. The STS is defined as (South African Department of Mineral Resources, 2016):

[*A*]n average change in hearing of 10 dB or more at frequencies of 2000 Hz, 3000 Hz and 4000 Hz in one or both ears as compared to the employee’s milestone baseline audiogram. (n.p.)

Although a much better classification method than the PLH, it gives little guidance on what steps are to be taken other than reporting to the employer and counselling the employee. This method is very similar to that adopted by the US Occupational Safety and Health Administration (OSHA) which also uses a standard threshold shift (OSTS). The major difference lies in the option for age adjustment of hearing levels using standard tables to account for age-related hearing loss (OSTS-A). In addition, the US method provides specific instructions on steps to be taken when OSTS is detected. The National Institute for Occupational Health and Safety (NIOSH) advocates what it calls a significant threshold shift (NSTS), defined as a 15-dB change from baseline at any test frequency. The method discounts correction for age and requires a third audiogram to confirm the STS. In terms of steps to take when an STS is detected, the NIOSH method provides clear guidance including retraining of employees on noise hazards, the use of HPDs and relocation to a quieter work environment. A study comparing the NSTS, OSTS and OSTS-A methods in terms of hearing impairment detection showed a higher prevalence for the NSTS than the other two methods. The prevalence using the OSTS and OSTS-A methods yielded 36% and 74% less detections, respectively, than the NSTS method (Masterson, Sweeney, James, Themann, & Wall, 2014). Despite all its merits, the NSTS is only a recommendation and not a legal compliance requirement. This means employers are at liberty to choose ^the OSTS method that is less onerous and is a statutory^ requirement. In addition, its indiscriminate use of any test frequency reduces its specificity towards identifying ONIHL. In the same vein, an alternative method uses the temporary threshold shift (TTS) that occurs immediately after exposure to predict the likelihood of a significant threshold shift (McBride, 2004).

The UKHSE developed a simple and practical method for categorising audiograms (Codling & Fox, 2017). This method utilises the sum of hearing thresholds at 1 kHz, 2 kHz, 3 kHz, 4 kHz and 6 kHz as compared to reference tables stratified by sex and age to determine the level of hearing (Musiba, 2015). The audiograms are ultimately categorised into acceptable hearing, mild hearing impairment, poor hearing and rapid hearing loss (as depicted in Table 1). This classification also sets levels for warning the employee of impending ONIHL or for referral to an ENT specialist (Table 2). This method provides a simple and straightforward means for the classification of audiograms, which comes complete with steps that should be taken in terms of hearing conservation. The age stratification, which is part of this method, has the advantage of taking presbycusis into consideration such that age-related hearing loss is not used to exclude older employees from gainful employment.

**TABLE 1:** UK Health and Safety Executive categorisation scheme based on sum of hearing at 1 kHz, 2 kHz, 3 kHz, 4 kHz and 6 kHz.

Category	Calculation	Action
1. Acceptable hearing ability Hearing within normal limits	Sum of hearing levels at 1 kHz, 2 kHz, 3 kHz, 4 kHz and 6 kHz. Compared with the figure given for appropriate age band and gender in standardised tables	None
2. Mild hearing impairment Hearing within 20th percentile, that is, hearing level normally experienced by one in five persons. May indicate developing symptoms of NIHL	Sum of hearing levels at 1 kHz, 2 kHz, 3 kHz, 4 kHz and 6 kHz. Compare with the figure given for appropriate age band and gender in standardised tables	Warning This means the employee has mild hearing impairment and needs training and counselling on how best to prevent further deterioration. No referral is warranted
3. Poor hearing Hearing within 5th percentile, that is, hearing level normally experienced by one person in 20. Suggests significant NIHL	Sum of hearing levels at 1 kHz, 2 kHz, 3 kHz, 4 kHz and 6 kHz. Compared with figure given for appropriate age band and gender in standardised tables	Referral This means that the employee has hearing worse than would be expected for his or her age and referral to an occupational physician, audiologist or an ENT specialist for further evaluation is indicated
4. Rapid hearing loss Reduction in hearing level of 30 dB or more, within 3 years or less. Such a change could be caused by noise exposure or disease	Sum of hearing levels at 3 kHz, 4 kHz and 6 kHz	Referral This means that the employee has hearing worse than would be expected for their age and referral to an occupational physician, audiologist or an ENT specialist for further evaluation is indicated. This type of deterioration could be because of noise or disease

*Source*: Health and Safety Executive. (2005). *Controlling noise at work. Guidance on regulations. The control of noise at work regulations 2005*. Norwich: Health and Safety Executive.

NIHL, noise-induced hearing loss.

**TABLE 2:** UK Health and Safety Executive categorisation according to age bands.

Sum of hearing levels 1 kHz, 2 kHz, 3 kHz, 4 kHz and 6 kHz				

Age	Males		Females	
	Warning level	Referral level	Warning level	Referral level
18–24	51	95	46	78
25–29	67	113	55	91
30–34	82	132	*63*	105
35–39	100	154	71	119
40–44	121	183	80	134
45–49	142	211	93	153
50–54	165	240	111	176
55–59	190	269	131	204
60–64	217	296	157	235
65	235	311	175	255

*Source:* Health and Safety Executive. (2005). *Controlling noise at work. Guidance on regulations. The control of noise at work regulations 2005*. Norwich: Health and Safety Executive.

The UKHSE categorisation scheme has come under great criticism for not utilising the characteristic notch at 4 kHz, which is regarded as an early sign pathognomonic of ONIHL. In addition, the criticism has arisen because of the use of a predominantly urban Caucasian reference population to formulate the classification; therefore, this scheme has limited generalisability (Cheesman & Steinburg, 2010). The justification for gender-based thresholds has also been criticised for not having a scientific basis (Cheesman & Steinburg, 2010). As much as this criticism may be valid, the notch and the shape of the audiogram are still available to the occupational physician or practitioner interpreting the audiogram. It would, therefore, be a matter of just a glance at 4 kHz to check for the presence of the characteristic notch in addition to the summation of the frequencies most affected by ONIHL. In short, all the data used to assess the results of audiograms in other categorisation or classification schemes are readily available on the audiogram for the sceptics to use to supplement the diagnosis. Limitations of the scheme for use in LAMI contexts such as Africa include the fact that no specificity or sensitivity tests have been conducted to compare it against other available methods in a predominantly ethnic group. In addition, because of the small number of occupational physicians, ENT specialist referrals may not be as effective as they are in developed countries such as the UK that has an abundance of such practitioners.

### The Tanzanian case

Although there is no doubting the fact that the most effective controls for ONIHL are higher up in the hierarchy of controls, and include substitution for quieter equipment, elimination of processes that generate excessive noise and automation, there are few studies evaluating the effectiveness of these controls (Morata & Meinke, 2016). The commonest controls utilised are HPDs. It is worth noting that the use of HPDs is affected by risk perception and comfort (Le, Straatman, Lea, & Westerberg, 2017). In addition, poor enforcement of legislation, coupled with more pressing priorities, leave workers exposed to noise levels that are detrimental to their hearing (Ologe, 2006). Audiometry, a health surveillance tool, can be used to motivate workers to protect their hearing and to invest in engineering controls. In order for audiometric results to be used in this manner, audiograms ought to be classified and data presented in a manner that allows both the employer and employee to make sound decisions. In 2015, the UKHSE categorisation of audiograms was used to determine the prevalence of ONIHL among miners in a Tanzanian mining company (Musiba, 2015). Prior to this, audiograms were not classified and therefore most cases of ONIHL were only detected during exit medical examinations. Once the cases were deemed to be occupational in origin, the affected employees received compensation and now had to deal with a life of disability. Using the UKHSE categorisation scheme, historical audiograms were classified and an NIHL prevalence of 47% was determined. The data were used to develop a procedure that required occupational health practitioners who conducted audiometry to classify audiograms immediately after audiometry so as to determine the next step in the care of at-risk workers. Workers exceeding the warning level were to receive in-depth counselling on noise as a hazard and the proper use of HPDs. Those who exceeded the referral limit were sent to an ENT specialist for examination and a diagnostic audiogram. Workers returning from an ENT visit would be required to go through a return-to-work process that manages the risk of ONIHL. This was to include audiometry at a frequency determined by the severity of hearing impairment and occupational hygiene measurements of their workplace. Individuals that were at high risk of worsening the severities of ONIHL were to be reassigned to work that significantly reduced the risk. To date, the procedure is firmly in place and has ensured the place for audiometry in the company’s hearing protection programme.

## Conclusion

Health surveillance through audiometry plays a key role in hearing conservation. The practice is beneficial only if it prompts the correct actions towards preventing NIHL. In order for audiometry to be effective, assuming that the technicians are competent and the equipment adequately calibrated, the audiograms need to be classified. The classification utilised ought to also be able to prompt occupational health practitioners to take the next vital step in preventing NIHL. Unfortunately, the majority of classification methods seem to be more suitable for compensation than for prevention purposes. The UKHSE audiogram categorisation scheme is simple and straightforward and requires basic addition skills. Furthermore, it prompts specific actions for every level of hearing threshold determined. Coupled with adequate training in the identification of noise hazards and the correct use of HPDs, the scheme can be a valuable tool in the prevention of NIHL. The method was used successfully in a Tanzanian mining company and continues to assist the early diagnosis and intervention of ONIHL.
